# Recent Interventions for Acute Suicidality Delivered in the Emergency Department: A Scoping Review

**DOI:** 10.5811/westjem.47474

**Published:** 2025-10-03

**Authors:** Katherine Dowdell, Michael P. Wilson

**Affiliations:** * Beth Israel Deaconess Medical Center, Department of Emergency Medicine, Boston, Massachusetts; † Carilion Medical Center, Department of Emergency Medicine and Psychiatry, Roanoke, Virginia

To the Editor:

We read with great interest the paper “Recent Interventions for Acute Suicidality Delivered in the Emergency Department: A Scoping Review” published by Hood et al in a recent issue of *WJEM*. We enthusiastically agree with the importance of emergency department (ED) expertise in the management of suicidal thoughts, as suicide prevention is a paramount public health concern. However, following careful reflection, we found methodological concerns regarding the exclusion of certain interventions and the manner in which conclusions were presented that warrant further discussion.

First, the authors excluded several ED interventions from this scoping review, including “screening, joint safety planning, patient education, lethal means counseling, follow-up contacts, and the involvement of friends and family.” The explanation for excluding these interventions is that they are “state-of-the-art practice (ie, generally accepted care)” as referenced in two textbooks, *Rosen’s Emergency Medicine: Concepts and Clinical Practice* and *Kaplan* and *Sadock’s Comprehensive Textbook of Psychiatry*.^1^,^2^ In reviewing the material cited from *Kaplan*, there is discussion of psychotherapeutic interventions and lethal means restriction as part of treatment for suicidal patients but no specific reference to these interventions being performed or evaluated in the ED setting.

The chapter cited from *Rosen* largely references “Caring for Adult Patients with Suicide Risk: A Consensus Guide for Emergency Departments” published in 2015.^3^ While this useful resource compiled best available information at the time, the authors explicitly state that the publication “is not intended to define a standard of care and should not be construed as one.” Excluding certain interventions based on documents that do not present a standard of care poses a danger of misrepresenting the evidence available in the field. As there are published papers regarding the excluded interventions within the time frame of interest and the existing data on implementation of these interventions in the ED setting is limited, inclusion of these interventions would have generated a more robust description and called forth a need for further research to generate evidence-based guidelines.

One additional aspect of the paper that raised concern was the manner in which conclusions were presented. The authors engaged in a qualitative synthesis of evidence without a critical appraisal of evidence. While the PRISMA-ScR checklist notes critical appraisal of individual sources as optional, completion of this process would have enhanced the rigor of this scoping review.^4^ The exclusion of this step may lead readers to a biased interpretation of available data. For example, in the paper on crisis response planning (CRP) by Bryan et al in 2017, the authors concluded that “CRP is highly effective for use in the acute care setting for acutely suicidal patients.”^5^ While this study is important, a closer look reveals considerable potential for bias. First, this was a small (n=32–33 in each arm), single-center study that exclusively recruited active-duty US Army soldiers, who were predominantly young, male, and White. Hence, the findings may not be readily generalizable to other care settings or populations. Second, a higher number of patients in the control group had a history of at least one suicide attempt, which may have biased findings away from the null. Third, there was substantial (nearly 20%) loss to follow-up, underscoring the potential for informative censoring. The totality of these observations limits rigorous interpretation of the study findings.

Taken together, the authors should be applauded for a much-needed summary of available data concerning suicide prevention in the ED. However, the article’s conclusion should be approached with caution. Evidence syntheses—should they be included in a scoping review—must be approached with the appropriate rigor and equipoise. Without this critical step, attention to important gaps in the field may be diminished.
